# Climate change: time to Do Something Different

**DOI:** 10.3389/fpsyg.2014.01294

**Published:** 2014-11-18

**Authors:** Nadine Page, Mike Page

**Affiliations:** ^1^Ashridge Business SchoolHertfordshire, UK; ^2^Department of Psychology, University of HertfordshireHertfordshire, UK

**Keywords:** climate change, pro-environmental behavior, behavioral flexibility, habit, awareness

## Abstract

There is now very little, if any, doubt that the global climate is changing and that this is in some way related to human behavior through unsustainable preferences in lifestyle and organizational practices. Despite the near conclusive evidence of the positive relationship between greenhouse gas emissions and global warming, a small proportion of people remain unconvinced. More importantly, even among the much larger number of people who accept a link between human behavior and climate change, many are inactive, or insufficiently active, in attempting to remedy the situation. We suggest this is partly because people are unaware both of how their day-to-day behaviors connect with energy consumption and carbon emissions, and of the behavioral alternatives that are available to them. This, we believe, is a key reason why individual lifestyles and organizational practices continue in an unsustainable way. We also suggest that the psychologists and behavioral researchers who seek to develop a better understanding of people’s relationship with, and reaction to, environmental issues, might also be on track to suffer a similar blindness. They risk becoming fixed on investigating a limited range of established variables, perhaps to the detriment of alternative approaches that are more practically oriented though, so far, less well explored empirically. In this article, we present the Framework for Internal Transformation as an alternative perspective on the variables that might underpin pro-environmental activity and behavior change. After briefly reviewing the related literature, we outline that framework. Then we present some early empirical data to show its relationship to a range of pro-environmental indices. We follow with a discussion of the framework’s relevance in relation to pro-environmental behavior change and make proposals for future research.

## INTRODUCTION

There is now very little, if any, doubt regarding the connection between human behavior, carbon emissions, and changes to the world’s climate. The scientific evidence surrounding climate change has grown considerably over the past three decades following the UN Conference on the Changing Atmosphere in 1988. More recently, a report compiled by an international consortium of scientists, the largest of its kind to date, has suggested that the large majority of climate researchers agree that human activity is contributing to global warming (Doran and Zimmerman, 2009; IPCC, 2013). To quote, the UN’s climate-panel scientists are 95% certain that humans are the “dominant cause” of global warming since the 1950s. Cumulatively, there is a clear message emanating from the scientific evidence: there is a significant relationship between changes to the climate and human behavior, as reflected in lifestyle preferences and organizational practices.

Despite the increasing scientific evidence it seems that a large majority of people still remain either unaware, in denial, or otherwise disengaged with the problem of climate change. This is reflected in the fact that UK energy consumption relating to transportation and households has continued to rise in recent years (DEFRA, 2006; notwithstanding some small drops recently, largely as a result of the financial recession). The IPCC (2013) suggests that human behavior is responsible for more than half of the observed increases in the climate. Elsewhere, it is also reported that only a minority of people are taking action to mitigate the effect (Whitmarsh, 2009). This means that for most individuals, daily life continues in a way that is unsustainable.

There are a variety of broadly psychological questions – that is, questions relating to human cognition, affect, and behavior – that arise in relation to climate change and sustainability. Do people have a realistic awareness of the scientific evidence showing the connection between human behavior in general and changes to the earth’s climate? If so, how does this make them feel and act? How aware are people of the effects of their own personal behavior and how this behavior might itself be unsustainable? In this regard, how aware are people of the alternative behavioral options that are available to them? And how willing and capable are people of changing their behavior in the cause of improved sustainability (explicitly acknowledging here the distinct difference between willingness and capability)? It is clear that these questions, and the answers to them, present a set of interrelated challenges to researchers who seek to better understand the relationship between people and their changing environment.

In this paper, we are not primarily interested in exploring pro-environmental behavior change in those who, notwithstanding the scientific near-consensus, are either skeptical or outright hostile to any posited link between human activity and climate change. According to figures from various sources (Spence et al., 2010; YouGov, 2013), around 5% of UK adults do not believe that climate change is a real phenomenon at all, with a further 20% or so believing that, while climate change is real, it is primarily caused by natural processes other than human activity. Lewandowsky et al. (2013a,b) have investigated members of this skeptical minority in relation, for instance, to their political views and to their concomitant rejection of other scientific consensuses (see also, Specter, 2009). The conclusions of Lewandowsky et al. (2013a,b) lead us to think it unlikely that a significant proportion of this minority will be susceptible any time soon to pro-environmental psychological interventions. (Indeed, any attempt along these lines is likely to be regarded as sinister and, if anything, to have the opposite of its intended effect.) For this reason, this paper focusses on how psychological interventions might assist the majority of people who accept anthropogenic climate change, and the closely related majority (55–60%, according to Thornton, 2009; Spence et al., 2010, respectively) who would like to do more to help the environment. Given the figures, we believe that any public policy would do well to have the same focus.

Among the majority who accept the existence of climate change and its relationship with human behavior, we suggest that one of the primary challenges for practical pro-environmental behavior change is a lack of attention and/or awareness, with this lack evident at two related levels. Dealing with attention first, people are very often sufficiently focused on activities related to their core proximal goals that they pay little or no attention to the environmental consequences of those activities, these being of secondary concern at best. For example, drawing on the second author’s experience of advising small companies on ways to cut their carbon emissions, the first challenge was often temporarily to draw attention away from the core business goal (i.e., generating products for market) and toward non-core aspects of a given business (such as the energy used for lighting and heating), aspects which nonetheless had a substantial effect on costs as well as environmental credentials. Without clients’ attention being so diverted, even temporarily, it was difficult even to begin a conversation about pro-environmental behavior. We have referred previously (Page and Page, 2011) to this fundamental attentional problem as being somewhat akin to what is called, in the perceptual and cognitive domain, ‘inattentional blindness.’ The mapping is not perfect, however, as wasted energy, say, is not literally present in the visual field. What is important though is to establish environmental concerns in the “attentional set” of the target audience for behavior change. Naturally, in circumstances where the consequences to an individual or organization of, for example, wasted energy are dramatic - such as when the cost of energy is extremely high – then there is an increased chance that attention is drawn to the otherwise unattended problem, in as much as the core goal (e.g., profit making) is directly affected. At present, it does not seem that increased carbon emissions are associated with sufficiently negative consequences to make them a sufficiently salient dimension of everyday activities. For example, for the richest decile of UK residents, the decile for whom personal carbon emissions are the highest, energy costs make up only 3.5% of their domestic expenditure, a percentage clearly insufficient to draw attention and to promote action (Vaze, 2009).

Second, regarding awareness, even once attention has been drawn toward issues of environmental concern, there is very often a lack of awareness/knowledge regarding the measures that could plausibly be taken to ameliorate the position. Taking again the example of a small business, one’s attention might be drawn to the high cost of lighting, but without both an awareness of potential alternatives and knowledge relating to the cost- and energy-saving consequences of each, significant behavior change is unlikely to occur. In previous work (Page and Page, 2011), we referred to this process of drawing attention to unattended problems and proposing viable solutions as the opportunities component of what we called the HOT topics of pro-environmental behavior change (Habits Opportunities Thoughts).

For people who are, in principle, open to change, another important aspect to consider is their personal belief that they are even capable of changing their behavior – an individual’s level of self-efficacy (Bandura, 1994). This is important, as the extent to which they believe that their specific efforts will be successful helps determine people’s behavioral motivation. Without sufficient self-efficacy, people might avoid attempting a specific behavioral change because they do not believe they are capable of its successful implementation. For this reason, we, among others (see below) suggest that a second key step in any intervention seeking to encourage pro-environmental behavior change involves developing intrinsic beliefs relating to self-efficacy so that people feel confident and empowered to take a different course of action.

It is not only “other people” who need to be attentive to alternative courses of action. Those psychologists and behavioral researchers who seek to develop a better understanding of pro-environmental behavior (and we include ourselves in this designation) might also need to broaden their attentional set. Based on a review of the literature on models of pro-environmental behavior and frameworks for pro-environmental behavior change (summarized below and reported in more detail elsewhere; see Page, in preparation), we suggest that a large majority of the theoretical and empirical research on pro-environmental behavior has become rather fixated on investigating, further investigating, and micro-refining a somewhat limited range of established variables, perhaps to the detriment of alternative approaches. Environmental researchers are, it seems, liable to become inattentive to novel approaches to behavior change in general, and to pro-environmental behavior change in particular.

With this in mind, we present below a preliminary investigation of a relatively novel framework for behavior change – called the Framework for Internal Transformation (FIT; Fletcher and Stead, 2000) – as it relates to pro-environmental action. In so doing, we organize the paper in the following way. First, we start by outlining some of the popular and empirically established frameworks relating to pro-environmental behavior that have placed emphasis on affective, cognitive, and behavioral dimensions. We consider the relative success that these psychological and behavioral approaches have enjoyed in the past. We then suggest that the net be cast rather wider in the search for psychological techniques that might usefully be applied in this domain and present the FIT Framework as a possible alternative approach that might have relevance in this domain. We then consider the applicability of FIT to pro-environmental behavior and present some preliminary empirical data. Based on the empirical insights offered by this early research, we consider the relevance of a FIT-based intervention for pro-environmental behavior change more generally.

### REVIEW OF PSYCHOLOGICAL APPROACHES TO PRO-ENVIRONMENTAL BEHAVIOR CHANGE

There are multiple and distinct types of pro-environmental behavior that have been considered from a variety of theoretical perspectives. These include environmental activism; non-activist public behaviors such as environmental citizenship or support and acceptance of public policies; private environmental activism; and environmental behavior in organizations (Stern, 2000). Several psychological theories and behavioral models have been designed specifically to explain the different types of pro-environmental behavior and efforts at pro-environmental behavior change [e.g., the Value-Belief-Norm Theory (VBN), Stern, 2000]. There are also other models of behavior that are more generic in nature and were first designed to explain behavior of other types, before being applied to pro-environmental activity [e.g., the Theory of Planned Behavior (TPB), Ajzen, 1991].

Regardless of their origin, the models each place a different emphasis on the factors that might influence pro-environmental behavior. Overall, they have often reflected two main types. The first type places greater emphasis on individual agency and the individual as the locus of behavior. From this perspective, behavior is perceived as an outcome of competing influences that are decided upon by the individual, typically in a balanced and rational way. Accordingly, behavior is largely determined by the strength of influence of an individual’s personal affective, cognitive, and/or behavioral characteristics and (perceived) competencies. In contrast, the second type of model is focused more on the social and physical context in which the behavior might occur. Approaches of this type place greater emphasis on the role of contextual and extrinsic factors that are, to a greater extent, perceived to be outside of individual control [e.g., Social Practice Theory (SPT), Hargreaves, 2011]. There are, of course, theories and models that sit astride these two camps and emphasize the interplay of both individual characteristics and contextual forces (e.g., the Comprehensive Action Determination Model; Klöckner and Blöbaum, 2010). However, despite such interactions being acknowledged in the theoretical frameworks that seek to explain pro-environmental behavior, we would argue that the significance of these interactions has often been underplayed in the models that seek to support pro-environmental behavior *change*.

Two popular psychological models of pro-environmental behavior that place greater emphasis on the role of individual characteristics are the Norm Activation Theory (NAT; Schwartz, 1977) and the VBN (Stern, 2000). The former was originally designed to explain altruistic and helping behaviors but in recent years it has been applied more widely to pro-environmental behaviors; this followed the conceptualization of such behaviors as moral acts that are determined by a sense of what it is right or wrong to do (Thøgersen, 1996). In contrast, the VBN approach was designed specifically to explain pro-environmental behaviors. In terms of their similarities, both the NAT and the VBN emphasize the influence of affect, values and beliefs in determining pro-environmental behavior.

According to the NAT, pro-environmental behavior occurs when people feel morally obliged to act in a given situation, based on the activation of a personal norm. The triggers to norm activation include: an awareness of the need for help; an awareness of the consequences of behavior; felt ascription of responsibility; and sufficient perceived behavior control to perform the action. Consistent with our previous characterization, the first two of these variables are dependent on an individual’s cognitive awareness not only of the need to act but also of the consequences of various actions. The third variable describes the strength of the affective relationship or a personal motivation to act, while the fourth can be described as an individual’s perception of their ability to act, as reflected in their perceived level of self-efficacy (Schwartz, 1977).

For the VBN, the strength of personal biospheric, altruistic, and egoistic beliefs underpins pro-environmental behavior. The VBN theory is a more inclusive framework than the NAT, as it identifies the attitudinal factors and personal capabilities of individuals, as well as the influence of contextual forces and habits. According to VBN, the causal chain starts with the strength of an individual’s core beliefs, values and norms, which determine an individual’s overall predisposition to act with pro-environmental intent. Three different types of value are identified: biospheric – a strong connection to the natural world; altruistic – a strong connection to other people; and egoistic – a strong connection to self. The strength of biospheric beliefs influences personal considerations with respect to the interconnectedness between human activity and the biosphere (cf. the New Ecological Paradigm; Dunlap et al., 2000). These might, in turn, lead to a personal motivation to avoid adverse environmental consequences, as is evident in the willing majority described above. However, in order to take action, an individual must feel that they are capable of worthwhile action, even though this perception might be at odds with their actual behavioral capabilities. Again, though, it is acknowledged that an individual’s attentional set and their personal level of self-efficacy are important in moving an individual from affect to action.

In addition to personal beliefs, the influence of habit is also acknowledged in the VBN theory (Stern, 2000). Habits are likely to have a significant impact on the degree to which people are aware and are capable of taking action. Research has shown that when behaviors become characteristic of habit, they are directed less by conscious awareness and intentions and more directed by cues in the context (Triandis, 1977; Ouellette and Wood, 1998; Bargh and Chartrand, 1999). Several pro-environmental behaviors have also been reported as having habit characteristics. For example, Danner et al. (2008) found that people who cycle regularly had highly accessible representations of cycling that were independent of their intentions to cycle. As such, behavior was performed somewhat automatically and relatively independently from cognitions. As well as influencing behavior directly, habits can also suppress the consideration of alternative behavioral options. For example, in an earlier study, Danner et al. (2007) found that the mental accessibility of habits increases with repetition and goes hand-in-hand with an inhibition of competing alternatives. In essence, when behavior becomes guided by habit, the behavior itself can happen automatically rather than intentionally, with alternatives barely considered.

The considerable amount of empirical research that has explored pro-environmental behavior and behavior change from the perspectives of the NAT and VBN theories has suggested that these models are more limited in explaining repetitive pro-environmental behaviors such as travel mode choice and recycling behaviors (see Stern, 2000; Hunecke et al., 2001; Harland et al., 2007). It appears, again, that pro-environmental behaviors can be guided more by habit rather than intention, perhaps helping to account for the value-action gap that is often reported in pro-environmental behavior research (Blake, 1999).

The role of habit in determining behavior has also been largely overlooked in models of behavior that emphasize the influence of cognitions. The TPB (Ajzen, 1991) is one of the most popular cognitive models of behavior. It was first developed to explain personal health behavior but has more recently been developed and applied to the domain of pro-environmental activity (see Donald et al., 2014). The model specifies three antecedent determinants of behavior, which have an indirect effect on behavior through their influence on behavior intention. The determinants are: attitudes toward the behavior, which reflect beliefs about the behavior and an evaluation of its expected outcomes; subjective norms, which reflect beliefs about the perceived social pressure to perform or not perform the behavior and an individual’s motivation to comply; and perceived behavioral control, which reflects beliefs about one’s capability and control to perform the behavior. The model suggests that favorable attitudes and subjective norms, coupled with perceptions of behavioral control, lead to strong behavioral intentions and, in turn, behavior.

The TPB model has been successfully applied to a range of different pro-environmental behavior intentions for both direct behaviors such as recycling (Cheung et al., 1999) and transport mode use (Donald et al., 2014), and indirect behaviors such as environmental activism (Fielding et al., 2008) and, more specifically, opposition to wind farm development (Read et al., 2013). It has also been successfully applied to pro-environmental behavior intentions in the workplace (Fielding et al., 2005; Greaves et al., 2013) though, overall, organizational settings have warranted far less empirical research and might pose different challenges for pro-environmental behavior compared with those encountered in a home context. In essence, the TPB is well supported in the pro-environmental domain. It has received and continues to receive, extensive empirical support.

The TPB is, however, not wholly without limitations. One problem concerns the ability of the model to predict actual behavior rather than behavioral intention. In line with the original model specification, much of the empirical research to date has explored the predictive value of the TPB toward behavioral intention rather than to behavior itself. The two are not the same. The predictive value of the TPB is substantially weaker for behavior compared to behavioral intention. In a meta-analytic review, Armitage and Conner (2001) found a 12% difference, from 27 to 39%, in explained variance between behavior and behavioral intention. As intimated above, the presence of habits is one factor that might account for this disparity. People do not do what they intend to do simply because the strength of habit relating to existing behaviors cannot be overcome by intentions alone. As with the value-action gap, habits might go some way to accounting for the intention-action gap that is also regularly reported in pro-environmental behavior research (Klöckner and Blöbaum, 2010).

Aside from the potential intervention of habit between intention and action, another limitation of TPB toward pro-environmental behavior research is the absence of a truly practical method for supporting people to *change* their behavior. TPB is a more useful model for understanding the variables that determine behavior than it is a framework for supporting pro-environmental behavior change. Two models that offer greater opportunities for better understanding and encouraging people’s susceptibility or resistance to changing behavior are the Stages of Change (SoC) model (Prochaska et al., 1992) and the more recent stage model of self-regulated behavior change (SSBC; Bamberg, 2013).

The original SoC model defines behavior as being positioned at one of five stages that are temporarily ordered and qualitatively different. These reflect an individual’s ‘level of motivational readiness’ (Heimlich and Ardoin, 2008, p. 279). The five stages are: (1) *pre-contemplation* – the individual is unaware of the problem and has no intention to change behavior; (2) *contemplation* – the individual is aware of the problem and is seriously considering changing behavior; (3) *preparation* – the individual is ready to change and is intending to take action; (4) *action* – the individual takes action to modify their behavior; and (5) *maintenance* – following action, the individual works to avoid relapse. Accordingly, a different intervention type is suggested for each stage, as each stage is likely to present a different challenge and hence to need a different approach (Nisbet and Gick, 2008). It is suggested that movement between stages is driven by two factors: an individual’s level of self-efficacy and their decisional balance, the latter reflecting the outcome of individual assessment of the pros and cons of particular behaviors (Armitage et al., 2004). It is possible to move backward and forward through the stages.

In a similar way, the SSBC model (Bamberg, 2013), which was designed specifically for pro-environmental behavior change, defines four distinct qualitative stages through which behavior change occurs. The first three of these stages are based on a foundation of cognitive awareness and intention. They are defined as: (1) *pre-decisional* – individuals consider competing wishes and turn some of these into binding goals to form a goal intention. Upon development of a goal intention the individual transits to the second stage, (2) *pre-actional*, whereby a specific behavior intention for guiding action is deliberated and decided upon. Next, formation of the behavior intention moves the individual to the third stage (3) *actional* and a narrowing down of behavioral options to a specific behavior, which is formulated through an implementation intention. Lastly, commitment to an implementation intention moves the individual to the (4) *post-actional* stage whereby the new behavior is performed (Bamberg, 2011, 2013).

Both the SSCB (Bamberg, 2013) and the SoC models (Prochaska et al., 1992) offer promising approaches to leveraging change in relation to pro-environmental behavior. A recent application of the SSCB model to a social marketing campaign showed greater effectiveness for encouraging car use reduction in comparison to a standardized information package (Bamberg, 2013). Both models acknowledge and consider the blindness (in our terms) that people might have toward the target problem and in relation to their current behaviors. In the SSCB model in particular, this is challenged directly by getting people to commit to a goal intention. We see this as a significant advantage over the other models of behavior that we have described so far. The SoC model also explicitly acknowledges the importance of self-efficacy for supporting people to move through the different stages.

Although both the SoC and SSBC models offer promising insights into the encouraging of pro-environmental behavior change, others (see Morris et al., 2012) have suggested that they might be too egoistical in their approach, in as much as the models miss or underplay the influence of structural economic, environmental, and social factors that could also influence performance of pro-environmental behaviors. An alternative approach offered by the SPT (see Shove, 2010; Hargreaves, 2011) places greater emphasis on extrinsic factors and how these might influence pro-environmental behavior and behavior change. In particular, the SPT theory considers how behaviors are embedded in the structures of everyday life, in the routine performances of social practices such as cooking, driving, washing, and shopping. From this perspective, behaviors, whether they are pro- or anti-environmental, become embedded within social practices (Warde, 2005) and, in turn, are perceived by people as being “normal ways of life” (Shove, 2004, p. 117). Through the exposure and repetition of social practices in day-to-day life, behavioral sets develop and become associated with different practices. Subsequently, behaviors are no longer determined by an individual’s personal competencies and intentions (as per NAM, VBN, TPB), but individuals become, instead, ‘carriers’ of social practices and thus become ‘performers’ of the behaviors that are required by the practice (Reckwitz, 2002). In essence, people develop behavioral habits and routines that are congruent with their environmental circumstances. As a consequence, people’s awareness of their actions might be lowered, a factor that we identified above as a key barrier to behavior change. The structure that embedded social practices offer might, of course, be advantageous once pro-environmental behaviors are established. In essence, the relatively stable routinization of daily life enables people to perform pro-environmental behaviors somewhat automatically, without overexertion of conscious decision-making processes.

Empirical research has supported this characterization. Transport choice is perhaps the best researched of the areas in which automatic action is cited as a negative environmental factor; specifically, the near automatic favoring of the private car over public-transport alternatives is frequently attributed to the force of habit (e.g., Dahlstrand and Biel, 1997; Verplanken et al., 1997, 2008; Klöckner and Matthies, 2004; Davidov, 2007). One can imagine, too, that the unnecessary turning on (and leaving on) of lights or of heating systems, the disposal (rather than recycling) of waste, or the unnecessary use of water, would all be under the influence of habits, rather than being driven by the more rational consideration proposed in the NAT (Schwartz, 1977), VBN (Stern, 2000), and TPB (Ajzen, 1991) theories. Again, habits are not necessarily environmentally deleterious: one might equally well be in the habit of cycling to work, turning lights off and dutifully recycling. Nonetheless, the environmental problems with which we are currently faced suggest that these ‘good’ habits are not yet the norm and that environmentally deleterious habits pervade.

### HABITUAL THINKING

So far in this article we have identified attention/awareness, self-efficacy, and behavioral habits as important considerations in the field of pro-environmental activity. We have suggested that both individuals and researchers might be prone to inattentional and habitual action, and that this might suppress their abilities to engage effectively with the problems that climate change presents. Up to now our attention on habit has been focused on behavior: we have considered how behavioral habits might support or impede pro-environmental activity in day-to-day life and have considered how habits might separate action from values, affect, and cognition. Next we briefly consider the process of habit development and how this might impede or support pro-environmental activity. In so doing, we further consider the connection between cognition, affect and behavior, and describe how cognitions and affect can themselves be habitual in character.

As well as their influence on behaviors, habits can also manifest in the way people think and feel. For example, in relation to the purchase of environmentally friendly detergents, Dahlstrand and Biel (1997) identified seven key steps in the development of an environmentally benign habit. These were: (1) activation (i.e., attending to the environment as a value); (2) attending to present behavior; (3) consideration of alternative behaviors; (4) planning new behavior; (5) testing new behavior; (6) evaluation of new behavior; and (7) establishment of new habit. Alongside each of these steps, they postulated factors that could either impede or promote progress at that point. It is notable that the first of Dahlstrand and Biel’s (1997) seven steps comes under the general heading of attentiveness and supports the notion that many people simply do not attend to, and are hence not aware of, their behavior. This lack of awareness could certainly impede attempts to change behavior, particularly as people under its influence might never progress to the latter stages of the new-habit-forming process. For the third step (the consideration of alternative behaviors), Dahlstrand and Biel (1997) also identified ‘negative beliefs about alternatives’ as an impeding factor, while the presence of ‘evident, existing alternatives’ was considered a promoting factor at this level. In this model, therefore, beliefs potentially constrain both the range of alternative behaviors that are considered and the way in which those alternatives are conceived.

In a previous discussion of the role of beliefs in behavior change (Page and Page, 2011), we drew a parallel with the role that beliefs are considered to play in therapeutic settings informed by Cognitive Behavioral Therapy (CBT) theory. CBT theory is influenced by the Greek philosopher Epictetus, who famously wrote that, “Men [sic] are not disturbed by things, but by the view that they take of them.” In the CBT conception, it is not events in the world that directly cause emotional or other disturbance. It is, instead, the thoughts/beliefs that an individual has about those events that intervene between events and feelings, and play a causal role in affecting the latter. In relation to automatic beliefs in pro-environmental behavior change, there is, we suggest, an analogous situation: the activation of just one negative environmental thought is enough to block entirely a change in behavior, even if that thought represents a cognitively distorted perspective on the world. For this reason, we suggest that close attention to thoughts, and in particular those negative thoughts that leap automatically to mind, is likely to be a necessary component of successful pro-environmental intervention.

We have highlighted the influence of cognitive, affective, and behavioral habits on pro-environmental behavior and behavior change. We propose that researchers need to take more serious consideration of the constraints that habits and behavioral inflexibility place on human action and the possibilities of change, this to include a willingness on the part of the researchers themselves (ourselves) to exhibit more flexibility in their (our) own investigations. In the spirit of this recommendation, therefore, in what follows we consider the FIT framework (Fletcher and Stead, 2000) as an alternative model of behavior that is explicitly accompanied by an associated framework of behavior change. By way of contrast with the models described above, FIT is a model that does, to a certain extent, consider the potential impact of cognitive and behavioral habits on pro-environmental behavior and behavior-change efforts, by explicitly measuring behavioral flexibility. The FIT framework has not been tried and tested in relation to pro-environmental activity, in the way that many of the preceding approaches have been. Nonetheless, we are currently undertaking empirical work to assess its value in this field. There follows a description of the FIT behavioral framework and our first empirical evaluation of its relationship with pro-environmental behavior.

### THE FIT FRAMEWORK

The FIT framework (Fletcher and Stead, 2000) comprises a collection of psychometrically validated tools (principally, the FIT Profiler; Fletcher, 1999) and a variety of behavioral interventions (principally, a Do Something Different programme). At its inception FIT, which is an acronym for Framework for Internal Transformation, was proposed as a framework to understand personal effectiveness in decision-making and behavior. It was offered as a theoretical framework for understanding the differences between people in how they cope with the situations they encounter (Fletcher and Stead, 2000). In line with this, much of the early research on FIT was focused on personal strain levels (e.g., see Fletcher, 1991). FIT is formulated around a framework of cognitive and behavioral competencies and strengths that, it is suggested, guide an individual’s perceptions of different situations and the demands that associate with these. In particular, the framework focuses on five cognitive competencies; also named Constancies, and the degree of flexibility across 15 behavioral dimensions, termed behavioral flexibility. These dimensions are perceived significantly to influence an individual’s decision-making processes and their execution of behavioral choices.

Framework for Internal Transformation theory acknowledges that behavior and thinking-style can both be prone to inflexibility and habit. Unlike other models of behavior, FIT emphasizes the idea that for maximum effectiveness one would not want to be located at any given point along a particular behavioral dimension. Instead, it is suggested that individuals should be comfortable operating at widely dispersed points along the dimension, so as to display the flexibility that is required to cope effectively and efficiently in different circumstances. To give an example, using a dimension of Introversion–Extroversion: FIT theory emphasizes that rather than seeking to locate one’s “character” at a particular point along this dimension, the varying demands of the real world would recommend flexibility, that is, sometimes being introverted, sometimes extroverted, as the occasion requires. The enhancement of such flexibility is intended, therefore, to counteract habitual, unaware behavior.

The FIT Profiler (Fletcher, 1999) measures the 15 behavioral dimensions defined by the FIT framework, and specifically measures the degree of flexibility in each (see **Table 1**). It also measures the five cognitive Constancies (Awareness, Balance, Conscience, Fearlessness, Self-Responsibility; see **Table 2**), in acknowledgment that thinking-style too can be prone to inflexibility and habit. The behavioral dimensions are defined by Fletcher and Stead (2000) as providing the ‘blueprint’ that allows individuals to behave effectively and flexibly across different situations. The behaviors are not considered as fixed traits but as competencies that are trainable and can be developed. The FIT Profiler measures such behavioral flexibility and directs its development.

**Table 1:** Dimensions of Behavioral Flexibility measured by the FIT Profiler.

Pole 1		Pole 2	Pole 1		Pole 2
Assertive	V	Unassertive	Behave as I wish	V	Behave as others expect
Conventional	V	Unconventional	Systematic	V	Spontaneous
Cautious	V	Trusting	Open-minded	V	Single-minded
Predictable	V	Unpredictable	Extroverted	V	Introverted
Energetic/Driven	V	Calm/relaxed	Definite	V	Flexible
Reactive	V	Proactive	Lively	V	Not lively
Group orientated	V	Individually orientated	Gentle	V	Firm
Risk taker	V	Cautious			


**Table 2:** Cognitive Constancies measured by the FIT Profiler.

Constancy	Item
Awareness	Are you always clear as to why you did something or are you often surprised?
Balance	When at work is your mind on other things?
Conscience	Do you believe you have to tell lies to succeed?
Fearlessness	Do fearful feelings stop you from doing things you want to do?
Self-Responsibility	Do you feel in control?

Fletcher and Stead (2000, p. 22) suggest that most people will have a “comfort zone” on each behavioral dimension. It is likely that this will reflect personal preferences and the way in which someone typically behaves in a given situation; in other words, it reflects their habitual tendencies. FIT theory acknowledges that as well as identifying personal preferences in each of the cognitive and behavioral dimensions, it is also important to offer a framework to encourage behavior change and personal development. The purpose of the associated FIT-Do Something Different (DSD) intervention is to expand the size of personal ‘comfort zones’ so that these exceed the “discomfort zone” (Fletcher and Stead, 2000, p. 22). People might, therefore, be better equipped to behave appropriately and flexibly in accordance with circumstance, and as guided by their conscious cognitions (Constancies).

Fletcher and Stead (2000) describe the cognitive Constancies as underpinning action. Furthermore, they suggest that if the Constancies are aligned at similar levels, they are more likely to guide decision-making and behavior that is effective and accords with current circumstance and personalized goals, rather than simply being driven by force of habit. Like Behavioral Flexibility, the cognitive Constancies are described as trainable. They can be strengthened and developed. There are five Constancies (see **Table 2**) and it is plausible that each has a role to play in determining pro-environmental behavior and driving efforts toward pro-environmental behavior change.

Having high levels of Awareness, which by definition is the degree to which an individual monitors and attends to their external and internal world, can be thought of as an antidote to being a habit-machine. It is about being awake and monitoring internal and external states and using feedback to guide actions, thoughts, and feelings. In relation to pro-environmental behavior and change, we suggest that individuals who have a higher level of Awareness will also be more aware of issues relating to climate change, the degree of sustainability in their personal lifestyle, the impact of their current behaviors, and the possibilities of change to become more pro-environmental.

The Balance Constancy is described as the ability of people to ensure every aspect of life receives due care and attention so that each part, be it work, non-work or self, is in-sync and that no one dominates. Accordingly, a person who scores high on Balance is able to prioritize different aspects of their life and allocate cognitive and behavioral resources toward these in accordance with demand. In relation to sustainability, a person with a low level of Balance might compromise this aspect of their lifestyle, potentially resulting in aspects of pro-environmental activity being pushed aside and excluded in the cognitions or behaviors of daily life. In contrast, individuals with higher levels of Balance might consider issues relating to sustainability as a priority alongside others, with the consequence that pro-environmental behavior is more prominent in their daily life.

The third Constancy is Conscience. This is described as the moral compass for decision-making and behavior. Conscience allows people to differentiate right from wrong and act on doing the right thing. It might then follow that an individual with a high level of Conscience will endeavor to make every decision an ethically and morally correct one. Fletcher and Stead (2000) suggest that individuals who have a high level of Conscience will never compromise morals in order to achieve an external goal. By plausible extension, individuals with higher levels of Conscience might feel more connected with issues of sustainability and therefore become engaged, both cognitively and behaviorally, with pro-environmental activity.

The fourth Constancy identified in the FIT framework is Fearlessness. Negative emotions such as fear and anxiety have been identified as barriers to action in several models of health behavior (see, e.g., Beck, 1967). When fear is particularly high it can restrict many aspects of daily life and can skew rational thought and action; excessive levels of fear can cause phobias. Fear can often be the main driver of behavior and the decisions people make. The FIT framework conceives fear as the emotional limiter of behavior that keeps people within their comfort zones, doing the things they have always done. It acknowledges that the influence of fear on people’s actions, choices and decisions might be unconscious, or, if felt, to be too powerful to overcome. As a consequence, FIT suggests that people stick to ‘safe’ patterns of behavior. In contrast, Fearlessness is achieved when people, to a significant degree, disconnect emotion from decision-making. Fearlessness supports individuals to act outside of their behavioral comfort zone, in accordance with their personal wants and desires. A sufficient level of Fearlessness might be necessary to encourage people to embed pro-environmental activities into their lifestyles, particularly if there are external barriers such as social norms that run counter to the desired actions. Higher levels of Fearlessness might give people the confidence to experiment with new and different ways of behaving, without the fear of failure.

The FIT framework (Fletcher and Stead, 2000) describes the Self-Responsibility Constancy as the barometer for measuring the extent to which an individual takes charge of their life and accepts responsibility for their actions and the things that happen to them, regardless of factors outside of their control. It is suggested that an individual who is self-responsible, will shape his or her own world. They will not believe in luck and chance, nor will they blame external factors for the things that happen to them. Accordingly, an individual who is self-responsible takes an active role in shaping their world so that it suits them. In relation to sustainability, it might be that an individual’s level of Self-Responsibility will influence their felt level of personal responsibility to do something to reduce their environmental impact. Having a high level of Self-Responsibility might strengthen the level of personal responsibility that an individual feels toward climate change and their personal contributions to it. This, in turn, might help initiate pro-environmental action.

As described above, the strength of an individual’s Constancies and their degree of Behavioral Flexibility might be separately important for determining the level of pro-environmental activity in personal lifestyles. In addition, the connectedness between both dimensions might also be an important consideration. The FIT Framework (Fletcher and Stead, 2000) emphasizes a bi-directional connection between people’s cognitions and their behavior. It suggests that Constancies can guide effective and flexible decision-making behavior and, in return, the experiences that are encountered can, through behavioral feedback, help to develop the Constancies further. The strength of this bidirectional relationship, particularly the effect of actions on thoughts, has often been underplayed in other models of behavior and frameworks for behavior change.

As noted previously, the FIT framework suggests that the cognitive Constancies provide the foundation for action; they guide decision-making and behavior. As such, they can act as a direct or indirect target for behavior change interventions. A direct approach to change would seek to develop the strength of each Constancy in order to lever changes in behavior. This is the approach supported by most existing psychological models of behavior and frameworks for behavior change. However, as mentioned previously, this method does not always result in new patterns of behavior and can result in the thinking-action gap often seen (Klöckner and Blöbaum, 2010). An alternative approach would be to change cognitions indirectly by developing behavior. According to this perspective, which subsumes an action-oriented approach, behavior is targeted directly to leverage indirect changes in thinking. The behavior change approach supported by the FIT framework, historically called DSD, uses both an indirect and direct approach to support behavior change. We suggest that in as much as it simultaneously targets cognitions and behavior (i.e., behavior independent of cognitions), the FIT framework might be a useful alternative approach for pro-environmental behavior change.

The FIT framework and DSD approach are novel perspectives that have received limited empirical examination. The empirical work that has been undertaken has mainly focused on health-related outcomes such as stress, weight loss, and family functioning (see Fletcher et al., 2011; Sharma, 2011). As far as we are aware, this is the first empirical study to explore the relationship between FIT variables and pro-environmental activity. Therefore, before we describe in detail the behavior change framework associated with the FIT framework, we first need to establish if there any relationships between FIT variables and pro-environmental activity and, if so, what these might comprise. What follows, therefore, is a description of our first empirical research exploring the relationship between FIT variables and indices of pro-environmental activity.

Based on our preceding rationale, we expect positive relationships between each of the FIT Constancies and cognitive and behavioral measures of pro-environmental activity. We also expect a positive relationship between Behavioral Flexibility and pro-environmental activity. This is because individuals who are more behaviorally flexible are likely to be more capable of adapting to new challenges (such as climate change) and of developing behavioral responses to mitigate their impact.

## MATERIALS AND METHODS

As this is the first empirical study that seeks to explore the relationships between FIT variables and pro-environmental activity, we thought it would be useful to identify the relationships in a sample that was as diverse as possible. With this intention in mind, we used an online survey to capture the relationships in a cross-section of participants that was obtained through a convenience sampling method. All respondents volunteered and gave informed consent to participate in the research. They were assured of the anonymity of the data they provided. The online survey was composed of several scales (as described below), and although they were not uncomplicated, responses suggested that respondents had understood the instructions. The survey was open for ∼2-months during which time 431 respondents started the questionnaire and 325 completed it in full. This equated to a 75% completion rate overall. Due to the possibility that the non-completing respondents later returned to the questionnaire and started to complete it a second time, data for the non-completers were removed. Therefore the results are based on a sample of 325 respondents.

### RESPONDENTS

Respondents in the study were 325 individuals [*n* = 87 (27%) male; *n* = 237 (73%) female], ages ranged from 17 to 71 years (*M* = 28.36, SD = 11.81). Two hundred and twenty four (69%) respondents were of white-British origin; 58 (18%) were Asian; 12 (4%) were Black; 7 (2%) were Chinese; 7 (2%) were of mixed race; and 8 (4%) other ethnicities. Regarding job type, 157 (48%) respondents were studying or in education; 47 (3%) were in administrative/secretarial roles; 86 (26%) were in professional roles; 22 (7%) were in managerial roles; 7 (2%) were self-employed; and 5 (2%) were unemployed or not working. One person did not report their type of work. Regarding highest educational qualification, 6 (2%) respondents had a PhD; 37 (11%) had an MSc degree; 108 (33%) had a BSc degree; 162 (50%) had A-Levels; and 11 (4%) had GCSEs. One person reported no educational qualifications. Respondents were self-selecting volunteers to the research and formed an opportunity sample.

We have described in detail here the characteristics of the sample. This is to give an indication of the diversity that was present. These demographics were not used to make smaller group comparisons in the inferential analyses; this was not the intention of this exploratory research.

### MATERIALS

The scales in the online survey measured dimensions of pro-environmental activity, personal FIT levels, and demographic information, as follows.

#### Indicators of sustainability

We used three separate scales to measure respondents’ cognitive and behavioral engagement with pro-environmental activity. The three scales were systematically connected such that they each separately assessed respondents’ pro-environmental thinking and behavior on a similar range of pro-environmental activities. We designed the survey in this way so that comparisons could be made between scores on the pro-environmental thinking and behavior scales, and between reported performance of pro-environmental behavior in different contexts.

The *pro-environmental thinking* scale measured cognitive aspects of sustainability across 37 items by asking respondents to rate the importance of a range of everyday pro-environmental behaviors for protecting the environment (e.g., “recycling materials”) on a 7-point Likert scale from 1 (extremely unimportant) to 7 (extremely important). Higher scores on the scale (minimum = 37; maximum = 259) represented stronger cognitive engagement with pro-environmental activity, that is, respondents were aware and thought that a larger number of the behaviors were important for protecting the environment.

The *home pro-environmental behavior* scale measured how frequently respondents performed pro-environmental behaviors in a home context. The scale was composed of 27 items, all of which matched to the scale item on the pro-environmental thinking scale. The items were measured on a 6-point scale from 0 (never) to 5 (always). Higher scores on the scale (minimum = 0; maximum = 135) represented greater engagement with pro-environmental behaviors in a home context.

The *work pro-environmental behavior* scale measured how frequently respondents engaged in pro-environmental behaviors in a work context. The scale was composed of 24 items, which were, where possible, matched to items in the home pro-environmental behavior and pro-environmental thinking scales. The items were measured on a 6-point scale from 0 (never) to 5 (always). Higher scores on the scale (minimum = 0; maximum = 120) represented greater engagement with pro-environmental behaviors in a work context.

In consideration of the influence of habit on behavior, we thought it useful to include two scales to measure pro-environmental behavior separately in home and work contexts. This allowed us to explore whether there were any significant differences between pro-environmental activities at home and work.

#### The FIT Profiler

A short version of the FIT Profiler (Page and Fletcher, 2006) was used to measure personal levels of FITness. The shorter scale was chosen to reduce the overall length of the online survey and to align with the exploratory nature of the study. The full version of the FIT Profiler is 75 items and would have significantly added to the survey completion time. The items included in this shortened version were determined by a psychometric report produced by Page and Fletcher (2006). The scale was composed of 20 items, 15 items measured Behavioral Flexibility and five items were used as key indicators for each of the cognitive Constancies.

The *Behavioral Flexibility* scale measured the degree of flexibility in an individual’s behavioral repertoire. The scale was composed of 15 bipolar items. Each item measured a different dimension of behavior as described by the FIT framework, e.g., “proactive vs. reactive,” “extroverted vs. introverted” (see **Table 1**). To complete the scale, respondents were instructed to indicate their range of behavior (i.e., the size of their behavioral repertoire) on each item on an 11-point scale. The response scale for each item represents the two extremes of the behavior in question, with nine intermediate points. Respondents who are behaviorally flexible will indicate a large behavioral range across all of the scale items. This would be shown by a response that spans from one end of the scale to the other, encompassing all 11 points. In contrast, respondents who are less flexible will indicate a narrower response that is typically situated at one end of the scale. The size of the range is recorded for each item and this reflects an individual’s degree of flexibility on the respective behavioral dimension. An overall Behavioral Flexibility score is computed as a percentage from the range scores of the 15 items (minimum = 0; maximum = 100); a higher score indicates a larger repertoire of behaviors, hence more Behavioral Flexibility.

The cognitive Constancies of Awareness, Balance, Conscience, Fearlessness, and Self-Responsibility were each measured by a single item. The item used for each was determined in a previous study that identified the psychometric properties of the FIT Profiler (Page and Fletcher, 2006). The strongest loading item for each Constancy scale was used in this shortened version of the scale. The Constancies were measured on a 0–10 scale with higher scores equating to higher levels of Awareness, Balance, Conscience, Fearlessness, and Self-Responsibility. A total score of the individual Constancy scores was computed to reflect the strength of FIT thinking. This is called FIT Integrity. Higher scores show higher levels of each Constancy (minimum = 0; maximum = 10) and overall FIT Integrity (minimum = 0; maximum = 100).

The psychometric report produced by Page and Fletcher (2006) identified the psychometric properties of the FIT Profiler in a sample of 1325. The results demonstrated good internal consistency for the cognitive Constancies: Cronbach’s alpha values ranged from lowest 0.67 (Self-Responsibility) to highest 0.87 (Fearlessness); FIT Integrity = 0.87; and Behavioral Flexibility = 0.91. The test-re-test coefficients ranged from 0.40 for Balance to 0.89 for Overall FIT (a combined score of FIT Integrity and Behavioral Flexibility).

#### Biographical and lifestyle questions

Respondents indicated their age; gender; ethnicity; highest education qualification; work/education status; and work/education hours. This information was collected as background data to inform the characteristics of the sample. It was not used to make smaller group comparisons in the inferential analyses; this was not the intention of this pilot research.

### DATA PREPARATION

Before proceeding with the data analysis we first acknowledge the self-report nature of the data and the possible implications of this. A self-reported survey approach was selected for ease of capturing a diverse sample for this pilot research. However, as with any self-reported data collection method, there is the potential that respondents might bias their responses toward social desirability. The distortion might be more prevalent here because the survey focuses on pro-environmental activity, a topic that has been shown to be prone toward the influence of social desirability (Schwarz, 1999).

The second limitation of using a self-report approach is that the answers provided do not necessarily reflect reality. In other words, the responses that respondents provide, particularly in relation to behavior, might not be representative of how they actually behave (see Huffman et al., 2014). This might be because respondents are intentionally distorting their answers for social desirability or because, as our reasoning throughout the paper has suggested, people are simply unaware of some aspects of the way they behave.

To mitigate these effects where possible, our analyses explored the relationships amongst the scale variables using within-subjects correlation analyses. In addition, the pro-environmental thinking and behavior scales, which contained a different number of items, were transformed to percentage scales prior to data analysis. The results for each scale are presented on a 0–100 scale with lower scores indicating lower levels of the variable measured.

## RESULTS

### DESCRIPTIVE STATISTICS AND RELIABILITIES

Considering the exploratory nature of this research and the novelty of the scales used, the first step was to check the descriptive properties and reliabilities of the scales. **Table 3** presents the alpha coefficient, mean, standard deviation, skewness, kurtosis, and other descriptive statistics for the pro-environmental activity and FIT Profiler scales. Overall, the alpha coefficients for the pro-environmental activity scales were highly satisfactory, ranging from 0.86 to 0.95 (*M* = 0.89). These results indicate substantial internal consistency of the scales. The scales also had acceptable (i.e., <1.00) levels of skewness and kurtosis, suggesting no serious deviations from normality.

**Table 3:** Descriptive statistics and reliabilities for the pro-environmental and FIT Profiler scales (*N* = 325).

				95% CI							
Scale	α	M	SE	LL	UL	Mdn	Minimum	Maximum	SD	Skewness	Kurtosis
Thinking	0.95	69.10	0.71	67.71	70.49	68.00	5.00	100.00	12.73	–0.30	0.18
Home bvr	0.86	58.48	0.80	56.91	60.06	58.00	9.00	96.00	14.45	–0.11	–0.02
Work bvr	0.86	65.05	0.96	63.15	66.94	65.00	13.00	99.00	17.40	–0.37	–0.25
Integrity	0.42	56.19	0.82	54.56	57.82	56.00	16.00	98.00	14.92	0.01	–0.31
Awareness	–	6.18	0.14	5.90	6.47	7.00	0.00	10.00	2.57	–0.37	–1.07
Balance	–	4.30	0.15	4.01	4.59	4.00	0.00	10.00	2.65	0.26	–0.95
Conscience	–	6.89	0.18	6.53	7.25	8.00	0.00	10.00	3.30	–0.81	–0.73
Fearlessness	–	4.54	0.15	4.24	4.84	4.00	0.00	10.00	2.71	0.29	–0.90
S-Responsibility	–	6.09	0.12	5.85	6.33	6.00	0.00	10.00	2.17	–0.40	–0.56
B-Flex	0.89	19.92	0.80	18.37	21.48	17.67	1.00	69.00	14.23	0.86	0.47

*Thinking, pro-environmental thinking; Home bvr, home pro-environmental behavior; Work bvr, work pro-environmental behavior; S-Responsibility, Self-Responsibility; B-Flex, Behavioral Flexibility*.

Scores on the pro-environmental thinking scale were moderate. This indicates that respondents believe that many of the activities are important for protecting the environment. The empirical scores distributed well across the theoretical scale (minimum = 0; maximum = 100). The home pro-environmental behavior scores showed that, on average, respondents performed some but not all of the pro-environmental activities. The mean was situated just above the halfway position on the scale. Overall, the empirical scores distributed well across the theoretical scale (minimum = 0; maximum = 100). The work pro-environmental behavior scores distributed to the upper- but not lower-end of the theoretical distribution (minimum = 0; maximum = 100). This suggests that all respondents performed at least some of the work pro-environmental activities.

For the FIT Profiler, it was only possible to calculate alpha coefficients for scales composed of more than two items. The alpha coefficients for these scales were, in the main satisfactory, ranging from 0.42 to 0.89 (*M* = 0.75). The alpha value for FIT Integrity was low, however. This may well be expected when investigating psychological constructs (see Burch et al., 2008; Zibarras et al., 2008), especially when they are measured using a limited number of items (Rust and Golombok, 1999). As this scale was a reduced version of the 50 item scale, and was composed of items measuring different aspects of cognition, a lower alpha coefficient was not unexpected and should not be considered too concerning, especially considering the diversity and limited number of items included in the scale. The data had an acceptable (i.e., <1.00) level of skewness and kurtosis suggesting no serious deviations from normality.

The Behavioral Flexibility scores were low and situated toward the lower end of the theoretical distribution (minimum = 1; maximum = 100). The FIT Integrity scores were moderate and distributed evenly across the theoretical scale (minimum = 0; maximum = 100). The scores did not reach the upper- or lower-ends of the scale suggesting that no respondents had either very poor or very high levels of Integrity.

Respondents reported lower levels of Balance and Fearlessness compared to the other Constancies. The variability of the Constancies was similar, indicating that there were approximately equal variations for each. Overall, the Constancy scores distributed evenly across the theoretical scales (minimum = 0; maximum = 10).

The alpha coefficients and descriptive statistics did, in the main, confirm the data suitable for parametric inferential analyses.

### INTERCORRELATIONS

#### Indicators of sustainability

The relationships between the pro-environmental scales (pro-environmental thinking, home pro-environmental behavior and work pro-environmental behavior,) were positive, moderate-to-strong, and significant (see **Table 4**). However, they were not too strong, which suggests a degree of independence among the scales. The relationship between pro-environmental thinking and pro-environmental behavior differed according to context. A larger proportion of variance was explained by the correlation between pro-environmental thinking and home pro-environmental behavior compared with work pro-environmental behavior (52 vs. 30%). A William’s *t*-test for non-independent correlations was used to compare the difference in strength for each correlation and showed this difference to be significant [*t_obt_*(323) = 5.83, *p* < 0.05]. This suggests that the relationship between pro-environmental thinking and home behavior was stronger. The relationship between home and work pro-environmental behavior was also reliable (explained variance = 52%). This suggests that there was a degree of shared variance in pro-environmental behavior between contexts, but still a large proportion of variance that is not explained by people’s cognitions and thus is attributable to other factors.

**Table 4:** Intercorrelations amongst the pro-environmental and FIT Profiler scales (*N* = 325).

Scale	1	2	3	4	5	6	7	8	9	10
(1) Thinking	–									
(2) Home behavior	0.72**	–								
(3) Work behavior	0.55**	0.72**	–							
(4) Integrity	0.21**	0.18*	0.21**	–						
(5) Awareness	0.16**	0.09	0.07	0.53**	–					
(6) Balance	0.08	0.11*	0.09	0.52**	0.02	–				
(7) Conscience	0.20**	0.14*	0.19**	0.60**	0.27**	0.03	–			
(8) Fearlessness	0.03	0.12*	0.10	0.49**	–0.05	0.24**	–0.05	–		
(9) Self-responsibility	0.11*	0.06	0.08	0.64**	0.28**	0.18**	0.23**	0.26**	–	
(10) Behavioral Flexibility	0.05	0.01	0.01	0.08	0.10	0.06	–0.04	0.08	0.04	–

**p < 0.01; *p < 0.05.

*Thinking, pro-environmental thinking; Home bvr, home pro-environmental behavior; Work bvr, work pro-environmental behavior; S-Responsibility, Self-Responsibility; B-Flex, Behavioral Flexibility*.

#### FIT variables and sustainability indicators

The relationships between FIT Integrity and the pro-environmental thinking and pro-environmental behavior scales were positive and significant (*r* = 0.21). The relationships were weak and explained a small proportion of the variance (4.4, 3.2, and 4.4% for pro-environmental thinking, home behavior and work behavior, respectively). Further analysis of each Constancy showed that Awareness was positively related to pro-environmental thinking (*r* = 0.16) but not performance of pro-environmental behaviors. The Balance Constancy positively related to pro-environmental behaviors performed at home (*r* = 0.11). Conscience was positively correlated with all three pro-environmental indicators (for pro-environmental thinking, *r* = 0.20; for home pro-environmental behavior, *r* = 0.14; for work pro-environmental behavior, *r* = 0.19). Fearlessness was positively related to performance of pro-environmental behavior at home (*r* = 0.12). The Self-Responsibility Constancy was related to pro-environmental thinking (*r* = 0.11). There were no relationships between Behavioral Flexibility and either pro-environmental thinking or pro-environmental behavior whether at home or at work (*r* ranged from 0.01 to 0.05).

Following on, partial correlations were conducted to explore whether the relationship between FIT Integrity and pro-environmental behavior was direct or was mediated by strength of pro-environmental thinking. The relationships between FIT Integrity and pro-environmental behavior performed at home and work whilst controlling for pro-environmental thinking were found to be non-significant [*r_partial_*(235) = 0.02, *p* = 0.69; *r_partial_*(235) = 0.12, *p* = 0.07, respectively]. This suggests that the relationship between FIT Integrity and pro-environmental behavior was indirect and somewhat dependent on strength of pro-environmental thinking.

## DISCUSSION

This study was the first to undertake an empirical investigation of the relationships between FIT variables and pro-environmental activity. It was a preliminary study. We used the FIT framework (Fletcher and Stead, 2000), a non-conventional framework that has received rather less empirical investigation than many other frameworks relating to behavior and behavior change. By taking this approach we wanted to test the possible relevance of a new theory of pro-environmental action and identify the personal characteristics that might support individuals in engaging with sustainability issues both cognitively and behaviorally.

The limited empirical research that already exists has explored the FIT framework in relation to health-related outcomes, specifically weight loss, stress, and family functioning (see Fletcher et al., 2011; Sharma, 2011). The research presented here has begun to elucidate the value of the FIT framework in a very different and timely domain – engagement with pro-environmental activity.

Specifically, there were reliable relationships between the FIT Constancies and the measures of pro-environmental thinking and behavior. The relationships with pro-environmental behavior were mediated by an individual’s cognitive engagement with pro-environmental activity. In contrast, there were no relationships with Behavioral Flexibility. It is, however, noteworthy that the Behavioral Flexibility scores were low and did not extend beyond the mid-point of the scale.

The pattern of results tentatively indicates that personal FITness levels do relate to an individual’s cognitive and behavioral engagement with pro-environmental activity. The mediated relationship between the FIT Constancies and pro-environmental behavior suggests that sustainability is both a psychological and a behavioral problem. In relation to these results, we suggest that those models that seek to explain pro-environmental behavior, and those that seek to encourage pro-environmental behavior change, should consider both the cognitive and behavioral dimensions of sustainability simultaneously and equally, rather than placing significant emphasis on one dimension at the detriment of the other.

In some ways, the FIT framework does allow us to look at both the cognitive and the behavioral characteristics that might relate to pro-environmental activity. The relationships found in our preliminary study suggest that an individual’s cognitive Constancies are more influential than their degree of Behavioral Flexibility in determining current pro-environmental behavior. This does not mean, though, that being behaviorally flexible is unimportant or that behavior change approaches should only focus on developing people’s cognitive engagement. Specifically, we have only shown here that Behavioral Flexibility does not correlate with established patterns of pro-environmental thinking or behavior. In retrospect, we perhaps should not have been too surprised by this. It may just indicate that people behave fairly habitually, whether or not their habits are pro-environmental. Indeed, the low distribution of Behavioral Flexibility scores suggests general patterns of behavior are fairly fixed. If this is the case more generally, then it will be important to focus interventions on enhancing the Behavioral Flexibility of the habitually “non-green,” leaving the habitually “green” to continue in their largely sustainable behavioral routines.

We have already discussed how habits can support pro-environmental behaviors. It seems counterintuitive to disrupt the behavior patterns of those individuals who are habitually sustainable in their approach, simply to make them more flexible. They have, after all, established patterns of behavior that are pro-environmental. What we are suggesting, therefore, is that a sufficient level of Behavioral Flexibility might be more important for supporting individuals to change behavior to become more pro-environmental, than it is for them to be pro-environmental *per se*. This would suggest that enhancing Behavioral Flexibility might make it easier to turn non-green behaviors to green, a hypothesis that deserves further investigation. The purposeful development of Behavioral Flexibility might be a necessary pre-cursor to support individuals who are habitually non-green toward a more pro-environmental disposition and more sustainable behaviors.

Whether, in the pursuit of more sustainable behaviors, it will be necessary to target interventions at enhancing the cognitive Constancies, is an open question. Although enhanced cognitive Constancies were associated here with more pro-environmental thinking and behavior, it is just as possible that a change in behavior can prompt a change in thinking, as vice versa. The FIT framework (Fletcher and Stead, 2000) emphasizes the bi-directional relationship between people’s cognitions and their behavior, and the DSD behavior change approach associated with the FIT framework seeks to direct development in both areas.

The FIT-DSD approach targets cognitions and behaviors both directly and indirectly through a structured change process based on habit reversal and (new) habit rehearsal. The indirect approach of the DSD intervention is characterized by an action-oriented stance in relation to change; it works by targeting behavior directly to leverage indirect changes in thinking. Specifically, the DSD approach encourages people to experiment with new behaviors, to try new and different ways of behaving in order to become more flexible. This, it is suggested (Fletcher and Stead, 2000), helps to expand the size of the behavioral repertoire. Through experimenting with new behaviors, people might be better equipped to weaken their existing habits (characterized by Fletcher and Stead as “habit-webs”) and also encounter new experiences that could challenge current thinking (see Page and Page, 2011).

In consideration to the powerful role of habit in cognition and behavior, and in separating behaviors from cognition (Klöckner and Blöbaum, 2010) and intention (Armitage and Conner, 2001), the FIT-DSD model of behavior change targets behavior directly by getting people to act out new behaviors rather than getting them to think about performing these. This might help to overcome (by rendering them irrelevant) the value-action gap (Blake, 1999) and the thinking-action gap that are so often evident in efforts toward pro-environmental behavior change. At the same time, therefore, the FIT approach challenges the ‘thinking trap’ (Fletcher and Pine, 2013) of researchers who tend to overestimate the power of thinking and hence to underestimate the power of actions.

There have been some practical applications of the FIT Framework to a range of psychological and social outcomes including stress, weight loss, and family functioning (Fletcher and Stead, 2000; Fletcher et al., 2011; Sharma, 2011). Taking weight-loss as an example, the DSD approach invites participants to engage in a structured program of doing something different on a regular basis for a set period of time. Across the time period of the intervention, the focus is on the development and performance of new behaviors. Importantly, these habit reversal and habit rehearsal tasks are not necessarily focused on the behavior that is the target of change. For example, in a weight-loss intervention, there is no necessity for all, or for even a majority of, the novel behaviors to have anything to do with food and exercise. The driving credo is that habits are not independent from one another, but exist in a mutually supporting network of habit-webs (cf. Neal et al., 2006) and routines. By breaking down the distal habits (the fixed routines of daily life) that form the habit-web in which the proximal target habits (e.g., overeating) reside, the DSD programme seeks to enhance generic flexibility. It seeks to put people into a (psychological) place in which they can change anything about themselves, before attempting to change any particular habit. As such, it comprises behavioral experiments at a generic level, designed to reinforce the belief that flexibility and change are a defining feature of a true comfort zone.

But why might this approach be an appropriate alternative model of behavior and a practical framework for pro-environmental behavior change? In many ways pro-environmental behavior change presents a very different challenge for the FIT Framework and the DSD approach from those to which it has previously been applied. One obvious distinction concerns the personal relevance of the target outcome. It is easy to see why someone might subscribe to a target outcome that sees them losing weight, but less easy, perhaps, to see why someone who is not already cognitively predisposed would set sustainability as a target outcome. Another distinction concerns the number of different changes that are required to enhance the sustainability of individual lifestyles, and the required perseverance of these changes across multiple contexts and in the presence of different pressures, e.g., competing social norms. These characteristics make pro-environmental lifestyle change a particularly challenging goal on which to focus.

Based on the results of our preliminary research, there is the possibility that the FIT framework and associated DSD approach might offer a useful alternative perspective on pro-environmental action. The approach offered by the FIT framework is, by nature, generic, and has applicability to many different behavior types. It offers a different perspective on the personal characteristics that relate to pro-environmental activity and deliberately steers clear of some of the habits that researchers are starting themselves to develop in their efforts to come to a better understanding of pro-environmental behavior.

We are well aware that even this preliminary study is not without its limitations. As noted previously, the data were self-reported and included only individuals’ perceptions of their environmental activities and levels of Behavioral Flexibility rather than objective measures. This raises potential limitations with regards to the accuracy of self-report data and the influence of self-serving bias (Schwarz, 1999), particularly in relation to the performance of sustainable actions in home and work contexts. Objective measures of both pro-environmental behavior and of Behavioral Flexibility are much more desirable and would, of course, offer a more reliable outcome (Huffman et al., 2014). It is, however, difficult to imagine a truly objective measure of Behavioral Flexibility that would not place enormous practical demands in terms of observing a given individual behaving in a variety of different contexts over time. The practicalities of such observation would render it unlikely that we could collect sufficient data to infer correlations and to analyze patterns in behavior. Objective measures of pro-environmental activities are more feasible, in as much as they can be extrapolated from proxy measures such as energy use, waste produced, travel modality and mileage, etc. However, as it was our intention to simply demonstrate the possible relevance of a new theory of pro-environmental action, then we think that self-reported data is sufficient in this instance. Many other studies in the area have employed similar methods (see Abrahamse et al., 2005; Osbaldiston and Schott, 2012).

We hope that the moderate sample size offers some reassurance that the results have a degree of validity and reliability. However, the relationships that we have evinced between FIT variables and pro-environmental activity are correlational rather than causal and, moreover, exhibit statistical relationships of only modest strength. We did not measure the relationships between alternative behavioral frameworks and theories and pro-environmental action, principally owing the large number of disparate measures that these alternative frameworks entail. We are not able to judge directly, therefore, the value of the FIT framework in comparison to other models. Nonetheless, based on these preliminary results, we can see that there is potential to explore further relationships between the FIT framework and pro-environmental activity in a more systematically comprehensive and validated way, and to consider further the value of the DSD approach in relation to pro-environmental behavior change. Our next steps, therefore, will be to undertake further empirical exploration of the relationship between FIT variables and pro-environmental activity in different samples and to compare these with the relationships between pro-environmental action and other psychological variables derived from alternative theories of behavior. By, we hope, confirming the statistical robustness of the relationships reported here, and by further exploring the value of the FIT framework in promoting practical action, we aim to encourage a degree of eclecticism in the psychological approaches to pro-environmental behavior change.

## Conflict of Interest Statement

The authors declare that the research was conducted in the absence of any commercial or financial relationships that could be construed as a potential conflict of interest.
